# Short-Term Hydrolytic Degradation of Mechanical Properties of Absorbable Surgical Sutures: A Comparative Study

**DOI:** 10.3390/jfb15090273

**Published:** 2024-09-20

**Authors:** Jakub Szabelski, Robert Karpiński

**Affiliations:** 1 Department of Computerization and Production Robotization, Faculty of Mechanical Engineering, Lublin University of Technology, Nadbystrzycka 36, 20-618 Lublin, Poland; 2 Department of Machine Design and Mechatronics, Faculty of Mechanical Engineering, Lublin University of Technology, Nadbystrzycka 36, 20-618 Lublin, Poland; 3 I Department of Psychiatry, Psychotherapy, and Early Intervention, Medical University of Lublin, 20-439 Lublin, Poland

**Keywords:** polymer sutures, mechanical properties, strength, elongation, modulus, tensile properties, biomechanics, wounds

## Abstract

Surgical sutures play a crucial role in wound closure, facilitating the tissue-healing process across various fields of medicine. The objective of this study was to analyse the impact of seasoning time during the initial days/weeks of seasoning in Ringer’s solution on the mechanical properties of five commercial absorbable sutures: SafilQuick+^®^, Novosyn^®^, MonosynQuick^®^, Monosyn^®^ and Monoplus^®^, each with different absorption periods. The results demonstrated that the SafilQuick+ and MonosynQuick sutures lost strength within 9–12 days, as evidenced by statistically significant changes in tensile strength. In contrast, the Novosyn and Monoplus sutures did not exhibit significant changes in strength during the study period. Statistical analysis confirmed significant differences in the behaviour of the individual sutures, highlighting the importance of selecting appropriate suture material in the context of the specific medical procedure.

## 1. Introduction

Surgical sutures are an essential medical material used mainly for closing wounds. Sutures are used daily in surgery for a variety of purposes. Their uses range from simple suturing of the skin after trauma or surgery, to internal surgery, where they are used to suture organs, to specialised applications such as ligatures, i.e., ligating blood vessels to stop bleeding [1,2,3]. They are also indispensable in cosmetic plastics and surgery, where special lifting threads are used. They are also used in dentistry to suture gums, in orthopaedics to connect muscle tissue or tendons, and in ophthalmology for eye surgery [4,5,6,7]. The key mechanical properties of suture materials play an important role in their overall function and appropriate selection [8,9]. The ideal suture material should not rupture unexpectedly during use or stretch when the wound swells, and it must be biocompatible, be easy to handle, form a secure knot [10], and, if used internally, biodegrade in due time [11,12,13,14]. Knowing the mechanical properties in terms of strength of different suture materials allows the first two characteristics to be assessed. Of the frequently mentioned mechanical properties of sutures, the basic strength properties are the most important [8,11,15].

Sutures are available in different types (due to their degradability), such as absorbable sutures, which dissolve, and non-absorbable sutures, made of different materials, allowing them to be tailored to specific requirements and tissues [16,17,18]. Absorbable sutures are designed to degrade in the body, eliminating the need for subsequent removal; they are mainly used in soft tissue surgery where long-term support is not required. Non-absorbable sutures, on the other hand, do not degrade and are often used in areas where permanent structural support is needed, such as in orthopaedics or plastic surgery [19,20]. The choice of the appropriate type of suture depends on the specific medical procedure and the type of tissue to be sutured [21,22,23].

Absorbable surgical sutures are the preferred choice for many medical procedures due to their ability to dissolve in the body’s tissues. Made from natural or synthetic polymers such as polyglycolic acid or polylactide, these sutures are designed to biodegrade gradually [4,24,25,26,27,28]. The time it takes for the sutures to be absorbed varies depending on the material and is adapted to the healing time of the tissue type in question, lasting from a few days to several months [29]. They are designed to minimise the inflammatory response, which promotes better wound healing. Resorbable surgical threads are broken down and absorbed by the body once the wound-healing process is complete. Degradation of implanted surgical suture polymer materials depends on a number of biological and physiological factors in the human body. Key among these is the biological environment, where pH [30] and enzymes can accelerate polymer breakdown. Inflammatory processes, triggered by the immune response, also contribute to degradation, particularly through cytokines and macrophage activity. This process begins with hydrolysis, where water penetrates the structure of the suture, breaking down the ester bonds in the synthetic polymers [31]. Natural threads, such as catgut, are degraded by proteolytic enzymes and phagocytosed by macrophages [32].

The presence of surgical threads triggers a local inflammatory response [33], attracting lymphocytes, macrophages and fibroblasts that help to degrade them. The degradation products are then absorbed into the bloodstream and excreted by the kidneys or metabolised in the liver. The resorption time depends on the thread material, the site of implantation and the patient’s condition. For example, catgut is absorbed within a few weeks, while synthetic threads can remain in the body for several months. Their tensile strength ensures that they will hold the connected tissues for the required time. These threads are mainly used in internal surgery, gynaecology, urology and general surgery, eliminating the need for subsequent removal [34].

Surgical sutures are graded according to their absorption time, which is crucial for surgical applications. Short-term absorbable sutures, typically absorbed within 30 days, are used in fast-healing areas such as skin or mucosal tissue. Medium-term sutures, which are absorbed in 30 to 60 days, are used in general and gynaecological surgery, where tissues need more time to regenerate. Long-term sutures, which are absorbed after more than 60 days, are preferred for procedures requiring long-term tissue support, such as orthopaedic or reconstructive surgery. Choosing the right type of suture is essential to ensure effective healing and patient safety [2,35,36,37].

The total absorption time is crucial in the long term, but as important is the strength changes of the suture during the initial absorption phase. Short-term absorbable sutures, although they may take one-and-a-half/two months to fully absorb, can undergo more than 50% weakening in as little as one week or so. Table 1 shows the expected times of loss of half of the initial holding power of different types of sutures [38].

Premature loss of the mechanical properties of absorbable surgical sutures can lead to serious complications such as wound dehiscence, which requires additional medical intervention [9,39,40]. It can also promote infection, as open wound edges create ideal conditions for bacteria. Insufficient tissue healing as a result of premature loss of thread support can result in unsightly scarring. As a result, patients may need additional treatments, increasing both the burden on the patient and the cost. Proper selection of threads and monitoring of the healing process are key to preventing these problems [38,41,42].

Due to the very important role of surgical sutures in the process of proper tissue healing, the authors aimed to investigate the effect of the seasoning time of typical commercially available surgical sutures on the degree of change in the basic mechanical properties due to suture degradation during the first post-implantation working period.

Seasoning under controlled conditions [43,44] allows for the simulation of the various scenarios that can occur in the body, enabling a thorough examination of how individual factors such as pH, enzymes, mechanical stress or temperature affect the durability of the material. Such initial research highlights the critical importance of understanding how sutures behave in the demanding conditions of the human body, where they are exposed to different fluids, varying pH levels, enzymes and other factors such as hydrolysis and elevated temperatures. Degradation of sutures under these conditions can significantly affect their mechanical strength and effectiveness during wound healing. By simulating these conditions, the study provides valuable information that can help in the selection of sutures that will maintain their integrity and function in such a demanding biological environment.

## 2. Materials and Methods

### 2.1. Materials

Five commercially available resorbable surgical sutures with different absorption periods were tested: two braided (multifilament) sutures with a fast absorption period, SafilQuick+ and Novosyn, and three monofilament sutures—two with a medium absorption period, MonosynQuick and Monosyn, and one with a slow absorption period, Monoplus. The sutures differ in their materials and typical applications. Only Monosyn and MonosynQuick have the same composition, but, according to the manufacturer’s declarations, MonosynQuick has been designed to be more hydrophilic, so that it absorbs water faster, accelerating the hydrolysis process that breaks down the suture material faster. At the same time, it has shorter polymer chains and lower crystallinity, which translate into faster degradation compared with Monosyn threads. The composition of the threads selected for the study, together with examples of fields of application, are shown in Table 2.

Samples were prepared by dividing the suture into 20 cm fragments. The manufacturer’s declared thickness of the threads was also controlled using a micrometer. For each sample group, 6–8 specimens were tested, as initial testing showed that this provided an acceptably low coefficient of variation between the results. Samples were seasoned in Ringer’s solution consisting of 8.6 g sodium chloride, 0.3 g potassium chloride, 0.33 g calcium chloride dihydrate per litre of water. This corresponds to the following electrolyte concentration: sodium—147 mmol/L, potassium—4 mmol/L, calcium—2.2 mmol/L, chloride—156 mmol/L [45,46]. The seasoning temperature was room temperature 20 ± 2 °C, as the study was planned as a pre-test. The seasoning of surgical sutures in Ringer’s solution prior to tensile strength testing is justified because of its isotonicity and biocompatibility imitating conditions in the body, while not inducing chemical reactions. Ringer’s solution perfectly simulates in vivo conditions, enabling the behaviour of sutures to be assessed in a realistic environment, leading to more reliable strength test results.

### 2.2. Tensile Strength Tests

The experimental tests were carried out using an MTS Bionix—Servohydraulic Test System testing machine (MTS Systems Corporation, Eden Prairie, MN, USA). Due to the small thickness of the material and the resulting high probability of crushing, the specimens were not placed directly into the grips of the testing machine, but were instead gripped using special grips designed to prevent mechanical damage to the specimen. When testing, tensile speeds were set according to the thread structure: 10 mm/min for faster-breaking multifilaments, 25 mm/min for monofilaments. The specimens were stretched until the threads broke, recording the force and strain of failure as well as the complete course of forces and strains throughout the strength test.

### 2.3. Statistical Analysis

The tensile strength results obtained were subjected to appropriate statistical analysis using the Statistica 12.5 software package. In order to identify discrepancies between the results obtained for a given suture, analyses were carried out by comparing multiple mean values, i.e., the strength and strain to break individual sutures, according to the successive seasoning periods of the suture. For this purpose, Tukey’s post-hoc test, the preceding tests of normality of the series distribution (Kolmogorov–Smirnov test, Lilliefors test and Shapiro–Wilk test) and tests of equality of variance (Fisher’s *F*-test, Levene’s test and Brown–Forsythe test) were used [47,48,49]. This approach is valid when multiple series need to be compared with each other, as the Tukey test provides control for the cumulative risk of type I error. With multiple Student’s *t*-tests, the risk of type I error (false positives) increases with each additional test. The Tukey test applies a correction that keeps the cumulative risk of type I error at the accepted level of α = 0.05. Multiple comparison tests allow for the identification of homogeneous subsets of means that do not differ significantly from each other. By performing pairwise comparisons, one can examine the differences between each pair of means and obtain a matrix where asterisks indicate group means with significant differences at an alpha level of 0.05. Several tests are available, including Scheffé’s test, Tukey’s test, the Newman–Keuls test, Duncan’s test and Fisher’s LSD test [50,51]. These tests vary in conservatism. A test is considered more conservative if it rejects individual comparisons less frequently than another test. In other words, a more conservative test has a lower ability to detect significant differences.

## 3. Results

**Figure 1:**
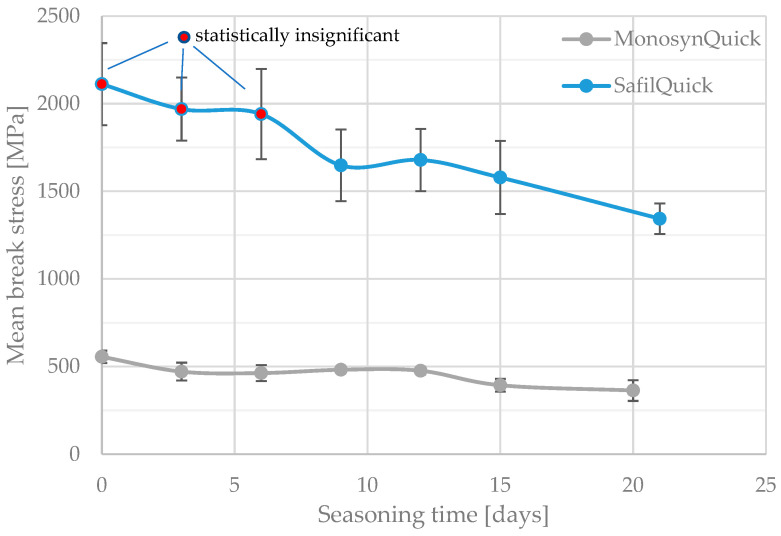
Mean values of failure stresses of tested fast-resorbable (Quick) sutures with increasing seasoning time in Ringer’s solution.

**Figure 2:**
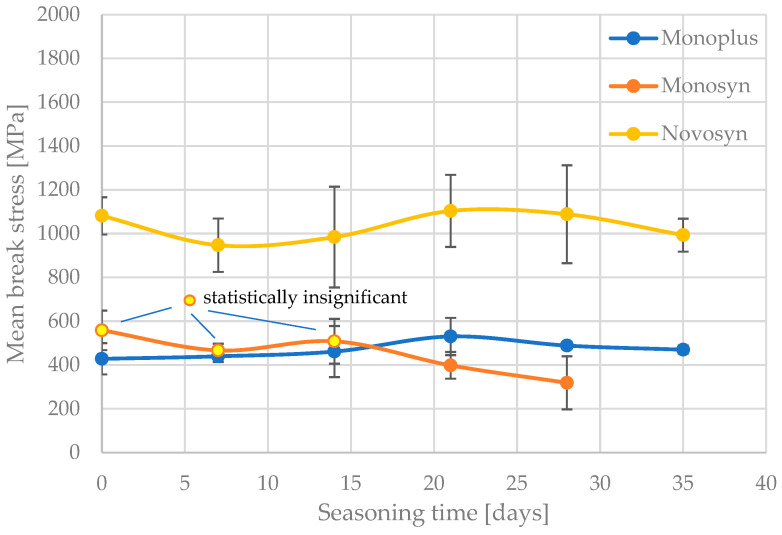
Mean values of the breaking stresses of the remaining resorbable sutures tested with increasing seasoning time in Ringer’s solution.

The results of the tests are shown in Figure 1 and Figure 2. They compile the mean values of the breaking stresses of the tested threads with the length of the seasoning time in Ringer’s solution, together with the standard deviation of the results within each series. The threads of the Quick (fast-resorbable) group and the other threads are presented separately.

On the basis of statistical analysis, groups with statistically insignificant differences (“X”) were separated for the individual threads analysed and the seasoning time, taking into account their tensile strength and deformation at break. Table 3, Table 4, Table 5, Table 6 and Table 7 show the results of this analysis. In addition, for better illustration, groups of points that do not show a statistically significant difference to the unseasoned samples have been marked with a different colour marker in Figure 1 and Figure 2.

Only the results obtained from statistical analysis reveal significant differences between the studied surgical sutures, in terms of changes in their mechanical parameters after the assumed seasoning period. The first two sutures, SafilQuick+ and MonosynQuick, stand out clearly, as they presented a statistically significant weakening of tensile strength after only 9–12 days of residence in solution. On average, a strength deterioration of approximately 30% was recorded after 3 weeks. It is interesting in this context to compare the changes in the average strain values of the strands to failure with the seasoning time. The stronger SafilQ showed a statistically significant reduction in mean strain after only nine days (by less than 10%), which even exceeded 1/4 after 3 weeks. The other braided suture, MonosynQ, behaved differently in this context, as no statistically significant change in strain to failure was shown during the analysed seasoning period, despite a deterioration in strength. Initially, such a change was observed in the mean values, but statistical analysis did not confirm it. It is worth noting that the aforementioned threads differed in their achieved strengths by a factor of approximately four—and SafilQ alone was the strongest of the threads tested.

In the case of Novosyn sutures, no statistically significant deterioration in tensile strength was recorded over 40 days of observation. There was a rather irregular change in the value of the mean strain to failure, but its variation could be explained by the scatter of the partial results obtained. In a similar manner, no significant changes in the strength of the Monoplus thread, which is half as strong, were observed. However, in its case, a significant elongation of strain to failure during seasoning was registered. Despite the high standard deviation values of the results obtained, a rolling elongation of the mean values was observed, with a statistically significant change shown at day 35.

By analysing the results obtained for the threads of the Monosyn group, a rather similar pattern of strength changes was observed with the degradation of the normal thread and the Quick version. An additional comparative, statistical analysis was therefore carried out directly between the aforementioned threads. Again, Tukey’s test was used. The five homogeneous groups of results are shown in Table 8.

**Figure 3:**
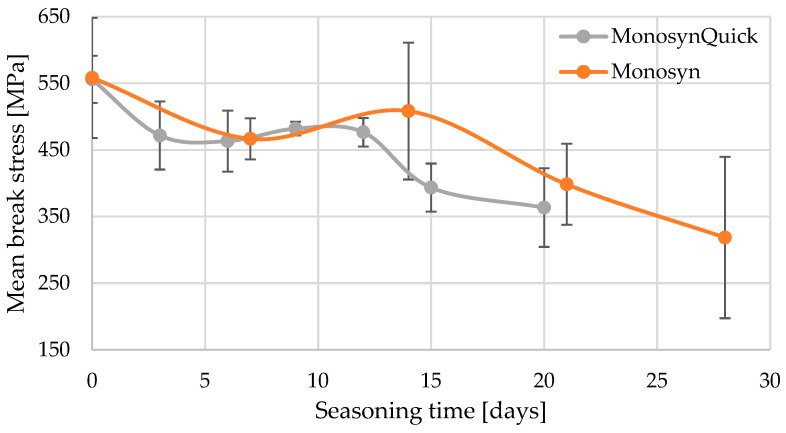
Mean values of the breaking stresses of Monosyn brand threads with increasing seasoning time in Ringer’s solution.

Unfortunately, it is difficult to draw a conclusion from the results of this analysis as to whether the Quick thread indeed undergoes faster absorption in the initial phase after use. Groups of homogeneous results overlap. The analysis is further hampered by the non-identical seasoning days on which both groups of threads were tested. The similarity in the change in strength of both threads can also be observed in the earlier graphs (Figure 1 and Figure 2) and Figure 3.

## 4. Discussion

The aggressive environment of the human body, which includes the presence of enzymes, moisture, varying pH, temperature, as well as contact with immune cells, significantly affects the degradation of polymeric biomaterials used for surgical sutures [52].

These biomaterials are exposed to hydrolysis, oxidation and enzymatic digestion, leading to their gradual degradation. High humidity and the presence of water in tissues can accelerate the hydrolysis of polymers, especially those containing ester bonds. Enzymes such as proteases and esterases can directly target the suture material, causing enzymatic degradation. In addition, inflammatory processes and immune reactions can cause oxidation of polymers, which also contributes to their degradation. For this reason, the design of biomaterials for surgical sutures requires consideration of these factors to ensure adequate strength and durability of the material until the sutures are no longer needed [31].

The surgical suture thread must be durable throughout the working period and especially during the initial period of overgrowth of the sutured wound. The experimental studies described in this thesis provide a general overview of the problem of early weakening of selected absorbable surgical sutures of different types and from different manufacturers due to the hydrolysis effect. Knowledge of the course of absorbed thread strength weakening is crucial for the surgeon when selecting the appropriate suture material for the individual patient and the wound the thread will be supporting [12]. A different suture will be selected in soft tissue surgery, where the thread must be strong enough to hold tension during suturing, but flexible enough to provide adequate wound protection. Yet another will be used in orthopaedics for suturing tendons, ligaments and during reconstructive procedures, where the thread must be strong enough to withstand the high mechanical stresses that can occur during movement and rehabilitation. Still another will be chosen in dentistry, where it is equally important that the thread is both strong and resistant to saliva and microorganisms.

The influence of the material composition and structure of the sutures on their mechanical parameters, such as tensile strength, elasticity and resistance to breakage, is crucial. The material composition of surgical sutures, i.e., the type of polymer used in their manufacture, has a significant impact on their mechanical performance [53]. For example, polypropylene sutures are extremely strong and flexible, which is beneficial in situations requiring long-term tissue support [54]. The effect of polymer type on the rate of absorption of absorbable surgical sutures is a key factor in determining their functionality in medical procedures. Absorbable surgical sutures are commonly used for wound closure, where a gradual disappearance of the suture with tissue healing is required. The different polymers used to produce these sutures have varying properties that affect the rate of absorption in the body. The most commonly used polymers are polyglycolide (PGA), polylactide (PLA), polydioxanone (PDS) and glycolic and lactic acid copolymers (such as Vicryl). Polymers with a higher molecular weight and higher crystallinity, such as PDS, have a slower absorption time, making them useful for situations requiring longer mechanical support. Sutures made from PGA, on the other hand, have a faster absorption rate, which is beneficial for rapid wound healing, but may be insufficient for tissues that need longer support.

The structure of the suture, i.e., how it is woven or manufactured, also plays a significant role. Threads can be monofilament (single strand) or multifilament (bundles/splices of threads) [55]. Monofilament threads have a lower surface area and are more resistant to absorption, which reduces the risk of infection [56]. Due to their structure, they can pass through tissue more easily, but may be less elastic and break under less stress. Multifilament sutures tend to have better elasticity and tensile strength, but their higher surface area can promote bacterial absorption and infection. As they are more susceptible to absorption of contaminants and moisture and exposure to ultraviolet radiation, that may result in faster degradation.

The effect of polymer type on the rate of absorption of absorbable surgical sutures is a key factor in determining their functionality in medical procedures. Absorbable surgical sutures are commonly used for wound closure, where a gradual disappearance of the suture with tissue healing is required. The different polymers used to produce these sutures have varying properties that affect the rate of absorption in the body.

Biodegradation of absorbable polymeric surgical sutures is a key process that enables their gradual elimination from the body once they have fulfilled their function. This process is mainly based on two mechanisms: hydrolysis and enzymatic degradation. Each of these mechanisms plays an important role in strand degradation and influences the breakdown of long polymer chains into shorter fragments that can be easily removed by the body’s cells.

Hydrolysis is the predominant biodegradation mechanism for most synthetic polymers, such as polyglycolide (PGA), polylactide (PLA) and polycaprolactone (PCL). This process involves the breakdown of chemical bonds in the polymer under the influence of water, which is present in the tissues. Water breaks the ester, amide or glycosidic bonds in the polymer chains, leading to their fragmentation. The rate of hydrolysis depends on the type of polymer, the strand structure and environmental conditions such as temperature and pH [57,58,59].

Enzymatic breakdown is particularly important for natural materials and some synthetic polymers. In this process, enzymes produced by the organism catalyse the breakdown of specific bonds in the polymers, accelerating their degradation. Natural materials, such as catgut, are more susceptible to this type of biodegradation, and the location of the strands in the organism and the presence of the relevant enzymes affect the rate of this process [60,61,62]. The degradation process is gradual and its rate depends on the chemical structure of the polymer and physiological conditions such as pH, temperature and the presence of enzymes. It leads to a progressive loss of mass and mechanical strength of the strands until they are completely absorbed by the body.

One of the reasons why changes in the strength of the two strands (Monosyn and MonosynQuick) were not distinguished may be precisely because of the environment in which the studies were conducted and, more specifically, the ambient temperature and the absence of enzymes, which, in the case of a fast-resorbable strand, may have a greater effect on decomposition than hydrolysis.

Given the greater savings in cost and time with absorbable sutures, they should be recommended as the primary suture material wherever possible. In experimental studies, there were no significant differences in postoperative satisfaction, incidence of postoperative wound infection or incidence of wound dehiscence between absorbable and non-absorbable sutures [22]. It is estimated that removing the need for sutures alone could save around £3 million per year in the UK by eliminating visits to the nurse. Patients would be able to manage their own wound care, resulting in additional savings [17], and it would be particularly desirable in paediatrics, for example, to avoid the need for suture removal in children, which can be painful and stressful, and in certain general surgery cases where the patient may have difficulty accessing post-operative care.

For the reasons outlined above, research into improving existing surgical threads is ongoing. Studies are concentrated in several directions, including towards the development of bioactive sutures. Traditional sutures are designed to be biologically inert, but no artificial material is completely biologically inactive. The need to improve wound-healing processes, especially in tissues with low regenerative capacity, is driving research towards developing suture materials that not only provide mechanical strength, but also support regeneration through an appropriate biological environment that promotes new tissue formation. Such sutures must ultimately fulfil four main goals: mechanical tissue approximation, support of new tissue growth, adequate suture degradation and good handling properties. They can be manufactured from biocompatible materials, minimising the risk of immunological reactions. Manufacturing of polymeric fibres at the submicron level allows imitation of the structure of the extracellular matrix. The suture surface can be designed with microscopic and nanoscale features that influence the integration of the implant into the tissue. The porosity of the material is crucial, as it must balance mechanical strength with the ability to infiltrate the tissue. Furthermore, the sutures can also serve as platforms for local drug release, which would minimise the risk of infection and ensure high local drug concentrations without side effects [4,16,63,64].

Other studies indicate that 3D FDM printing technology can be used to produce suture materials much cheaper than commercial ones, which could revolutionise the medical supply chain, especially in hard-to-reach places such as rural regions, war zones and space missions [65,66]. Research is being conducted into threads whose production will not cause a hazardous environmental impact due to the need for energy consumption, or the use of large quantities of toxic organic solvents or incompatible metal-based catalysts [67]. Sutures for external surgical applications, prepared from collagen derived from tannery waste and impregnated with a plasticiser, are also being tested [68].

The development of bioactive sutures involves extensive, multi-field research and its eventual introduction into surgical practice involves complex, lengthy and costly regulatory procedures [69]. Therefore, preliminary studies are not performed in an in vivo environment, but a corresponding artificial environment. The sutures tested in this study were analysed during seasoning in Ringer’s solution, which is designed to replicate conditions in the human body as closely as possible. However, the work showed that one of the threads that was supposed to be fast-resorbable was not significantly different from its non-fast-resorbable variety, which may be one of the limitations of the work. Estimating the actual strength of a thread based on its behaviour in a Ringer’s solution simulating real working conditions may not be precise enough for some threads. Therefore, in the future, it is planned to extend the study to include analyses carried out in a thermal chamber that additionally simulates human body temperature [70]. Additional testing may also include testing sutures in other environments, for example in saliva for sutures used in the oral cavity [71,72], in cerebrospinal fluid (CSF) for neurosurgery [73], in blood for vascular or cardiovascular procedures [74,75], in acidic gastric fluid for gastrointestinal surgery [76], in urine for sutures used in urological surgery, and in other environments [77]. In each of these environments, the physical and chemical impacts on the suture may be different and lead to a different degree of reduction in its strength. Furthermore, it is worth extending the tests to other methods of loading. In addition to static tensile loading, which although provides some information about the strength of the suture, cyclic, dynamic fatigue loading can be simulated, which would be closer to the actual working conditions of the suture used to close the wound [78,79].

## 5. Conclusions

The biodegradation of absorbable surgical threads is a complex process that depends on various factors. Both hydrolysis and enzymatic breakdown contribute to the gradual degradation of the suture, allowing it to be safely absorbed by the body. The speed of this process depends not only on the type of polymer and structure of the suture, but also on the physiological conditions of the patient, such as tissue pH, presence of enzymes and blood supply.

1. The study showed that absorbable surgical sutures experience a statistically significant reduction in tensile strength during the first three weeks after implantation.
2. Not all rapidly absorbable sutures weaken significantly during this critical initial period, but those that do may weaken wound stability.
3. These findings highlight the importance of suture selection based on specific clinical needs, as early loss of strength can affect the wound-healing process.
4. The choice of polymer in suture materials should be carefully matched to the procedure, site of application and patient condition to minimise the risk of postoperative complications.

The study provides key information on the degradation and mechanical performance of different suture materials. We demonstrated that both tested Quick sutures (Monosyn Quick and Safil Quick), as well as one non-Quick suture (Monosyn), experienced a statistically significant reduction in tensile strength during the first three weeks of conditioning in Ringer’s solution.

With further, extended research in the future, these findings may assist surgeons in selecting the most appropriate type of suture based on specific clinical needs. Surgeons should be aware of these degradation patterns to ensure that the chosen suture maintains adequate strength during the critical phases of wound healing.

## Figures and Tables

**Table 1:** Classification of sutures according to the period of tissue support.

Suture Group by Absorption Time	Estimated Retention Time of 50% of Initial Strength	Total Absorption Time
Short	5–7 days	42–56 days
Medium	14–21 days	60–90 days
Long	28–35 days	180–210 days
Extra-long	90 days	approx. 390 days

**Table 2:** Basic information on the suture materials analysed.

Suture Name	Total Absorption Time	Material	Sample Applications
SafilQuick+	~42 days	Polyglycolic acid	Gynaecology, urology, dentistry, paediatric surgery, ophthalmology ligation
Monosyn Quick	56 days	Glyconate (72% glycolide, 14% caprolactone, 14% trimethylene carbonate)	Plastic surgery, odontology and otolaryngology, gynaecology, urology
Novosyn	56–70 days	Copolymer of 90% glycolide and 10% L-lactide Poly (glycolide and L-lactide 90/10)	Gastrointestinal surgery, gynaecology, trauma and orthopaedic surgery, ophthalmology, urology, skin suturing
Monosyn	60–90 days	Glyconate (72% glycolide, 14% caprolactone, 14% trimethylene carbonate)	Gastrointestinal surgery, gynaecology, urology, skin suturing, ligature
MonoPlus	180–210 days	Poly-p-dioxanone	Abdominal anastomoses, orthopaedics, paediatric cardiac surgery, vascular surgery, neurosurgery, transplantology

**Table 3:** SafilQuick suture statistical analysis results—groups of homogeneous results.

Seasoning Time [Days]	No. of Specimens	Mean Break Stress [Mpa]	Break Stress SD [Mpa]	Groups of Homogeneous Results			Mean Strain at Break [mm]	Strain at Break SD [mm]	Groups of Homogeneous Results		
				1	2	3			1	2	3
0	8	2111.74	233.95	X			24.06	2.51	X	X	
3	8	1969.07	180.38	X			28.13	1.65	X		
6	8	1940.78	257.26	X	X		23.71	3.52		X	
9	8	1647.90	204.73			X	22.00	3.44		X	X
12	8	1678.79	177.89		X	X	21.81	2.21		X	X
15	8	1579.04	208.33			X	21.01	2.10		X	X
21	8	1343.68	87.54			X	17.74	1.07			X

**Table 4:** MonosynQuick suture statistical analysis results—groups of homogeneous results.

Seasoning Time [Days]	No. of Specimens	Mean Break Stress [Mpa]	Break Stress SD [Mpa]	Groups of Homogeneous Results				Mean Strain at Break [mm]	Strain at Break SD [mm]	Groups of Homogeneous Results
				1	2	3	4			1
0	8	555.94	35.51	X				36.33	10.89	X
3	8	471.60	51.02	X	X	X		36.20	7.21	X
6	8	463.24	45.84		X	X		36.25	2.92	X
9	8	482.01	10.20	X	X			37.63	1.90	X
12	6	476.52	21.30	X	X	X		38.82	1.38	X
15	6	393.53	36.08			X	X	31.46	5.49	X
20	7	363.49	59.02				X	29.62	6.37	X

**Table 5:** Novosyn suture statistical analysis results—groups of homogeneous results.

Seasoning Time [Days]	No. of Specimens	Mean Break Stress [Mpa]	Break Stress SD [Mpa]	Groups of Homogeneous Results	Mean Strain at Break [mm]	Strain at Break SD [mm]	Groups of Homogeneous Results	
				1			1	2
0	8	1081.18	85.03	X	37.79	4.85	X	
7	8	946.87	121.86	X	24.88	9.25	X	X
14	8	984.25	230.17	X	21.15	13.09		X
21	8	1103.74	164.40	X	28.77	9.36	X	X
28	8	1088.61	223.77	X	21.32	7.95		X
35	8	993.05	75.38	X	31.32	4.53	X	X
42	8	1010.79	85.03	X	32.83	4.85	X	X

**Table 6:** Monosyn suture statistical analysis results—groups of homogeneous results.

Seasoning Time [Days]	No. of Specimens	Mean Break Stress [Mpa]	Break Stress SD [Mpa]	Groups of Homogeneous Results			Mean Strain at Break [mm]	Strain at Break SD [mm]	Groups of Homogeneous Results
				1	2	3			1
0	7	558.21	90.27	X			40.28	16.27	X
7	7	466.66	30.72	X	X		36.05	11.92	X
14	7	508.28	102.63	X	X		40.62	12.51	X
21	7	398.41	60.73		X	X	34.69	9.37	X
28	7	318.52	121.06			X	31.29	9.40	X

**Table 7:** Monoplus suture statistical analysis results—groups of homogeneous results.

Seasoning Time [Days]	No. of Specimens	Mean Break Stress [Mpa]	Break Stress SD [Mpa]	Groups of Homogeneous Results	Mean Strain at Break [mm]	Strain at Break SD [mm]	Groups of Homogeneous Results	
				1			1	2
0	7	428.34	71.57	X	24.91	14.22	X	
7	7	439.76	25.79	X	26.58	6.98	X	X
14	7	461.58	116.72	X	33.61	11.68	X	X
21	7	530.19	85.27	X	34.09	4.96	X	X
28	7	488.60	16.44	X	38.11	10.61	X	X
35	7	470.07	19.27	X	45.95	4.27		X

**Table 8:** Summary of the results of the statistical analysis comparing the strength of two Monosyn thread variants.

Seasoning Time [Days]	Monosyn Variant	Mean Break Stress [MPa]	Break Stress SD [MPa]	Groups of Homogeneous Results				
				1	2	3	4	5
0	-	558.21	90.27	X				
0	**Quick**	**555.94**	**35.51**	**X**	**X**			
3	**Quick**	**471.60**	**51.02**	**X**	**X**	**X**	**X**	**X**
6	**Quick**	**463.24**	**45.84**	**X**	**X**	**X**	**X**	**X**
7	-	466.66	30.72	X	X	X	X	
9	**Quick**	**482.01**	**10.20**	**X**	**X**	**X**	**X**	
12	**Quick**	**476.52**	**21.30**	**X**	**X**	**X**	**X**	**X**
14	-	508,28	102.63	X		X		
15	**Quick**	**393.53**	**36.08**			**X**	**X**	**X**
20	**Quick**	**363.49**	**59.02**				**X**	**X**
21	-	398.41	60.73		X		X	X
28	-	318.52	121.06					X

The values of the Quick variant have been bolded for better readability.

## Data Availability

Data presented in this study are available from corresponding authors upon request.
